# Life expectancy, long-term care demand and dynamic financing mechanism simulation: an empirical study of Zhejiang Pilot, China

**DOI:** 10.1186/s12913-024-10875-7

**Published:** 2024-04-15

**Authors:** Xueying Xu, Yichao Li, Hong Mi

**Affiliations:** 1 School of International Studies, Zhejiang University, Hangzhou, China; 2 School of Public Affairs, Zhejiang University, Hangzhou, China

**Keywords:** Long-term care insurance, Life expectancy, Dynamic financing model, Simulation analysis, Zhejiang province, China

## Abstract

**Background:**

China has piloted Long-Term Care Insurance (LTCI) to address increasing care demand. However, many cities neglected adjusting LTCI premiums since the pilot, risking the long-term sustainability of LTCI. Therefore, using Zhejiang Province as a case, this study simulated mortality-adjusted long-term care demand and the balance of LTCI funds through dynamic financing mechanism under diverse life expectancy and disability scenarios.

**Methods:**

Three-parameter log-quadratic model was used to estimate the mortality from 1990 to 2020. Mortality with predicted interval from 2020 to 2080 was projected by Lee-Carter method extended with rotation. Cohort-component projection model was used to simulate the number of older population with different degrees of disability. Disability data of the older people is sourced from China Health and Retirement Longitudinal Study 2018. The balance of LTCI fund was simulated by dynamic financing actuarial model.

**Results:**

Life expectancy of Zhejiang for male (female) is from 80.46 (84.66) years in 2020 to 89.39 [86.61, 91.74] (91.24 [88.90, 93.25]) years in 2080. The number of long-term care demand with severe disability in Zhejiang demonstrates an increasing trend from 285 [276, 295] thousand in 2023 to 1027 [634, 1657] thousand in 2080 under predicted mean of life expectancy. LTCI fund in Zhejiang will become accumulated surplus from 2024 to 2080 when annual premium growth rate is 5.25% [4.20%, 6.25%] under various disability scenarios, which is much higher than the annual growth of unit cost of long-term care services (2.25%). The accumulated balance of LTCI fund is sensitive with life expectancy.

**Conclusions:**

Dynamic growth of LTCI premium is essential in dealing with current deficit around 2050 and realizing Zhejiang’s LTCI sustainability in the long-run. The importance of dynamic monitoring disability and mortality information is emphasized to respond immediately to the increase of premiums. LTCI should strike a balance between expanding coverage and controlling financing scale. This study provides implications for developing countries to establish or pilot LTCI schemes.

**Supplementary Information:**

The online version contains supplementary material available at 10.1186/s12913-024-10875-7.

## Background

The lack of sufficient long-term care (LTC) for older individuals has become a pressing concern in both developed and developing countries with global population aging and increased longevity [1]. Although healthy life expectancy generally increased over last decades [2], the episode of disability in older people could have catastrophic impact on their household welfare [3]. Several developed countries, such as the Netherlands, Germany, and Japan, have established social long-term care insurance (LTCI) to address LTC demands of households with disabled older individuals. This approach proves more efficient in pooling disability risks than private LTCI [4, 5]. Nonetheless, many developed countries had to reform their LTCI systems to deal with increasing aging population with LTC demands, often by raising premiums. Even though, these adjustments usually had time lags which affected the long-term sustainability of LTCI schemes. However, establishing social LTCI in developing countries proves more challenging than in developed countries because the lower income of residents restricts the financing capacity of LTCI. In addition, the lack of high-quality death registration and health survey data hinders optimizing LTCI systems design according to changing LTC demands, particularly in developing countries or small areas [6].

Massive evidence shows that there will be a steady and slow increase in life expectancy [7–9]. Evidence from developed countries shows that the long-term care needs increasing rapidly because of the increasing life expectancy [10, 11]. The trend of the gap between life expectancy and healthy life expectancy is still inconclusive [12], which also affects the identification of LTC needs [13]. There is still mixed conclusion of disability and LTC demands trend in the future based on the three different assumptions of health transitions [14–16]. Whereas, there is less evidence regarding the assessment of LTC needs under different mortality scenarios. Zeng, et al. [17] calculated long-term care needs under different life expectancy scenarios, but the setting of life expectancy was relatively subjective. Besides, many studies in country-level controlled the impact of underreported mortality on the LTCI system by using modified mortality data [18, 19], but few studies in the provincial level took that into consideration.

Most countries such as Germany and the Netherlands adopt a fixed percentage of income model to collect social LTCI premiums from individuals [20], and a few countries such as Singapore adopt a fixed amount premium model [21]. The premium of Germany LTCI has been 3.05% of gross income or 3.40% if individuals aged 23 and above without children since 2020 [22]. The Netherlands also has a tax-funded LTCI with the compulsory contribution of 9.65% of taxable income since 2017 [20]. In Singapore, fixed amount premium of LTCI is determined by the age of starting contribution and sex. The premium for a 30-year-old male (female) is around 200 (250) Singapore Dollars in 2020 [21], with an increase of 2% per year from 2020 to 2025 [23]. Financing parameters from both models should be adjusted regularly to ensure sustainability [24, 25]. In China, both models are adopted in different LTCI pilot areas [26], but the areas that adopt the fixed amount of premium have not increased the premium level since the pilot, which affects long-term sustainability.

OECD countries will face high pressure of LTCI financing because of increasing average public LTC expenditures to 2.3% of GDP in 2040 for the future financing level of LTCI [27]. Therefore, an adjustment factor is suggested incorporated to simulate LTCI fund to reduce future financing pressure [22], but a higher short-term financing will bring greater resistance to reforms. Most simulation studies on China’s LTCI, based on fixed percentage of income model, demonstrated that LTCI financing will increase rapidly based on different disability scenarios [28–31]. Some studies also simulated LTCI financing based on fixed amount of premium model [32, 33], but they did not consider its variation under different mortality scenarios. Only one study modified the mortality in a pilot city by using national mortality data when simulating the dynamic financing burden [34]. However, it only simulated to 2040 which did not cover plateau period of China’s aging.

China, as a developing country, pioneered social LTCI schemes in 2016. Local governments were granted significant autonomy, resulting in fragmented LTCI structures due to regional disparities in the pilot cities [35]. Thus it has become crucial to ensure the sustainability of China’s LTCI pilot areas. Zhejiang Province stands as a representative case among these pilot areas and its five cities (Tonglu, Ningbo, Jiaxing, Yiwu and Whenzhou) have piloted LTCI since 2017. Zhejiang has standardized disability assessments, coverage groups, benefit levels, and financing amounts of LTCI in province-level by 2022 [36]. It faces rapid aging ahead with high life expectancy in China. Notably, Zhejiang, one of the areas with fixed amount of premium of LTCI in China, has never increased its fixed premium since the pilot’s inception [36]. This lack of financing adjustment coupled with inflationary pressures strains Zhejiang’s LTCI fund. Zhejiang has capacities to facilitate LTCI operations through modified financing mechanism as the demonstration zone for the Initiative of Common Prosperity in China. Therefore, it can serve as a practical model for other developing countries establishing LTCI schemes to evaluate life expectancy and LTC demand parameters and guide its LTCI financing.

In summary, massive studies predict the LTC needs in developed countries and China. However, most of the studies on LTCI financing in China pilots overlook the potential death underreporting in census and uncertainty of mortality in projection period, which may misestimate the future LTC needs and financing pressure. In addition, current studies on the sustainability of China’s LTCI rarely involve the dynamic financing adjustment of fixed amount of premium model, and most studies do not cover the plateau period of China’s aging in the future, which may underestimate the financing level to achieve sustainable LTCI. Therefore, drawing from the Zhejiang Province case in China, this study proposes a dynamic financing mechanism to achieve a balance between sustainability and efficiency in social LTCI schemes, utilizing a simulation model with limited mortality and disability information. Our aim is to offer insights for developing countries to establish or pilot LTCI schemes. Three research questions will be addressed:

- What is the long-term trend of life expectancy in Zhejiang from 1990 to 2080?
- What extent of LTC demand will be reached among older people in Zhejiang from 2023 to 2080, with aging process?
- What level of LTCI dynamic financing standards will achieve an actuarial equilibrium of the LTCI fund in Zhejiang, with rising life expectancy and LTC demand?

## Methods

### Data sources

For demographic data, the age-specific mortality and the population number by gender are from population census of Zhejiang Province in 1990, 2000, 2010 and 2020. The population census, which has been conducted once every 10 years since 1990, is a complete account of the entire population, mortality and fertility by age and sex in each census year and has the province-level representativeness of Zhejiang. Child mortality data is from Chinese Center for Disease Control and Prevention (CDC) in 1990–2013 [37], and official annual data of Zhejiang reported up to 2020 [38]. Chinese CDC sorted and estimated under-5 mortality rates in China before 2013 with county-level and province-level representativeness, including data in Zhejiang. Data on the prevalence rate of disability of the older people is sourced from China Health and Retirement Longitudinal Study (CHARLS) in 2018. CHARLS is a national representative survey which covers a wide range of topics related to the adults aged 45 and above, including demographic information and health status. The national prevalence rate of disability by age and sex from CHARLS is used as a proxy for Zhejiang referring to existing research, due to lack of latest representative disability data in Zhejiang [39]. Older people are defined as those aged 60 and above based the statistical standards from World Health Organization [40], whose age groups are covered by CHARLS. The benefit criteria and financing criteria data is from the LTCI official regulations of pilot cities in Zhejiang [41–45]. Healthcare Consumer Price Index (CPI) from 2010 to 2020 in Zhejiang is from National Bureau of Statistics of China, covering the socio-economic indicators at province-level [46]. The change rate of total fertility of China from 2020 to 2080 is from World Population Prospects 2022 which forecasted fertility in country-level around the world [47].

### Estimation of mortality pattern with three-parameter model life table approach

Model life tables methods are widely used in simulation of mortality for their effectiveness and accessibility to overcome the limited mortality information in developing countries [48, 49]. Two-parameters log-quadratic model considering the child and adult mortality overcomes the shortage of Coale-Demeny and UN model life tables, among those model life tables methods [50]. Three-parameter log-quadratic model is designed on this to calculate the life table considering extra old-age mortality parameter with an adjustment of intercept with real census information [51]. It is so-called developing countries mortality database (DCMD) model which was adopted in the World Population Prospects 2019 since the three-parameter log-quadratic model life table was initially used in those developing countries without the high-quality mortality data [52]. The basic function of DCMD model is showed below:

1 $$\ln ({m_x})={\hat {a}_x}+{b_x} \cdot \ln ({\,}_{5}{q_0})+{c_x} \cdot {[\ln ({\,}_{5}{q_0})]^2}+{v_x} \cdot k$$

2 $${\hat {a}_x}=\left\{ \begin{gathered} {a_x},x<60 \hfill \\ {a_x}+\ln [\frac{{\ln (1 - {\,}_{{15}}{{\hat {q}}_{60}})}}{{\ln (1 - {\,}_{{15}}{q_{60}})}}],x \geqslant 60 \hfill \\ \end{gathered} \right.$$

This study used adjusted DCMD model to estimate the mortality in Zhejiang from 1990 to 2020 to make it usable for open population conditions. Child mortality (${\,}_{5}{q_0}$) is the first parameter of DCMD model, and adult mortality (${\,}_{{45}}{q_{15}}$) is the second parameter to be compared with estimated adult mortality (${\,}_{{45}}{\hat {q}_{15}}$) from two-parameter log-quadratic model with adjustment factor $k$. Specifically, child mortality by gender in consecutive years is estimated by sex ratio of child mortality in China [53]. Adult mortality in census years is calculated from census life table directly as the register completeness of adults’ death is higher in China [53]. Moreover, we averaged old-age mortality estimated from two-parameter log-quadratic model and from survival model for midpoint of old-age mortality between censuses (1995, 2005 and 2015) [51]. We averaged old-age mortality from two-parameter log-quadratic model and from census life table calculations for census years (1990, 2000, 2010 and 2020). The adjusted DCMD model was constructed on the incorporated old-age mortality. After that, the cubic hermite polynomial interpolation approach (pchip package in R) was adopted to estimate adult and old-age mortality from 1990 to 2020 [54]. The life table for consecutive years was estimated with DCMD model.

After that, Lee-Carter method extended with rotation (LC_ER) (mortcast package in R) was used to forecast the mortality up to 2080 [55], which provides critical death parameters to assess the LTCI demands in our case area. Since in low mortality countries, mortality decline is decelerating at younger ages and accelerating at older ages [56], the static assumption of mortality decline of traditional Lee-Carter model would be anomalous in long-term projection. LC_ER is a time-varying Lee-Carter model considering the changes of mortality decline between different age groups when modeling, which was widely recognized and adopted by World Population Prospects 2022 [57, 58]. Therefore, potential LTCI demands change caused by changes in old-age mortality decline in long-term projections could be captured by LC_ER. The predicted mean of life expectancy would be set as the medium life expectancy scenario, and the lower and upper 95% predicted interval would be set as the low and high life expectancy scenarios.

### Number of severe disabled older adults

LTCI beneficiaries refer to the severe disabled population according to the rules of LTCI in Zhejiang [36]. The study used the cohort-component projection (CCP) method to forecast the number of older population of Zhejiang from 2020 to 2080 [59]. The number of age-specific population by sex from Zhejiang population census 2020 was used as the base population of CCP model. Furthermore, the age-specific prevalence rate of disability from CHARLS 2018 was calculated. After that, the number of severe disabled older adults as the LTCI beneficiaries was calculated by multiplying age-specific older population and prevalence rate of disability. The basic project method is as follows:

3 $${\,}_{{x+1}}P_{x}^{{t+1}}={\,}_{x}P_{{x - 1}}^{t}\left[ {{L^t}(x+1)/{L^t}(x)} \right]+N{I^*}$$

${\,}_{{x+1}}P_{x}^{{t+1}}$ represents the population of single age groups with the age of *x* to *x* + 1 at the *t* + 1 time. $\left[ {{L^t}(x+1)/{L^t}(x)} \right]$ represents the survival ratio of age *x* to *x* + 1 at t time. $N{I^*}$ represents the net migration numbers in the corresponding age group from the *t* to *t +* 1 period, from other regions to Zhejiang.

Our estimated mortality will be used in CCP model. Since the total fertility of Zhejiang is lower than that of China, this study assumed that the total fertility of Zhejiang would start at 1.04 in 2020 based on Zhejiang population census [60]. Then, the future trend of Zhejiang’s total fertility would follow the United Nations’ estimated change rate of total fertility of China from 2020 to 2080 [47]. For net migration, The Census Survival Ratio Method was used to estimate the migration pattern based on the census data [61]. As one of the highest net in-migration provinces since 2010, Zhejiang will face the lower net in-migration intensity and be close to migration equilibrium in 2040 [62]. Based on this, it is assumed that the net migration rate in Zhejiang will experience a linear decrease and realize migration equilibrium by 2045.

Disability is defined as a difficulty in performing at least one of six Activities in Daily Living (ADL) [63], including bathing, dressing, eating, getting in/out of bed, using the toilet, and controlling urination and defecation in CHARLS. Then, mild disability is defined as having difficulty in 1–2 items of ADL, moderate disability as having difficulty in 3–4 items of ADL, and severe disability as having difficulty in at least 5 items of ADL [64, 65]. Based on the discussion on the Disease Expansion, Disease Compression and Dynamic Equilibrium Theory [66], three different scenarios in changing disability were calculated [16]: a 0.8% annual decrease for age-specific prevalence rate of disability as the low disability scenario, the constant age-specific prevalence rate of disability as the middle disability scenario, and a 0.8% annual increase for age-specific prevalence rate of disability as the high disability scenario.

### Dynamic financing actuarial model of social LTCI schemes

The study built a macro simulation model to further simulate the expenditure, financing and fund balance of LTCI based on the projection of severe disabled older population ($DisOP$) aged 60 and above and contribution population ($CP$) of LTCI aged 20 and above. The macro model is showed below:

4 $$\eqalign{& LTC{E_t} = \cr& ({\alpha _{\rm{1}}} \times HbdcCos{t_t}\, + \, {\alpha _2} \times IcCos{t_t} \cr & + {\alpha _3} \times HbdmcCos{t_t} \cr & + {\alpha _4} \times NhcCos{t_t}) \times DisO{P_t} \cr} $$

5 $$\eqalign{& Current\_Balanc{e_t} =\cr& premiu{m_{{t_0}}} \times {(1 + \lambda )^{t - {t_0}}}\cr & \times C{P_t} - LTC{E_t} \cr} $$

6 $$\eqalign{& Accumulated\_Balanc{e_t} =\cr & Accumulated\_Balanc{e_{t - 1}} \times (1 + \gamma )\cr & + Current\_Balanc{e_t} \cr} $$

In Formula (4), $LTCE$means LTC expenditures, $HbdcCost$, $IcCost$, $HbdmcCost$ and $NhcCost$ represent the unit cost of home-based daily living care (HBDC), institutional care, home-based daily living & medical care (HBDMC) and nursing hospital care per person per year, respectively. Among them, HBDC means that beneficiaries only receive formal daily living care services at home but without any medical care. HBDMC means that beneficiaries receive both formal daily living care services and professional medical care services at home. The difference of institutional care and nursing hospital care lies in that the former focuses more on daily living care, while the latter specializes in medical care. From 2023 to 2080, the unit cost of each type of LTC services is given an increase of 2.25% annually based on the average increase of healthcare CPI from 2010 to 2020. $\alpha$ means the percentage of different types of LTC services utilization. Formula (5) describes the dynamic financing model and current balance of LTCI every year. $premiu{m_{{t_0}}}$is the fixed amount of premiums of LTCI in our base period. $\lambda$ is annual growth of the amount of LTCI premiums. Formula (6) shows the accumulated balance of LTC fund which is determined by the current balance and the accumulated balance in previous period. $\gamma$is the interest rate of LTCI fund which represents the time value of the LTCI fund. Taking the inflation rate (2.25%) as a reference in the simulation process, we test the minimum value of $\lambda$ that ensures a consistently positive accumulated balance in the LTCI fund up to 2080 across various disability scenarios.

### Parameters of LTCI schemes in Zhejiang Province, China

The policies of LTCI schemes in five pilot cities in Zhejiang are sorted in Additional Table 1 (see Additional file 1) [41]. The LTCI schemes in Jiaxing City are representative among five pilot cities of LTCI in Zhejiang. Firstly, Jiaxing is the first city covering all employees and urban and rural residents equitably with the same benefits and premium since the adoption of LTCI (in 2017), which has navigated the reform of LTCI in Zhejiang. Secondly, LTCI benefits in Jiaxing are at the middle level among the five pilot cities, which is representative of average level in Zhejiang. The maximum benefits of HBDC in Jiaxing are lower than those in Yiwu and Wenzhou, and equal to those in Tonglu and Ningbo. Besides, the maximum benefits of institutional care are also lower than those in Yiwu, but higher than those in Tonglu and Ningbo. Overall, Jiaxing’s LTCI benefits stay average in Zhejiang. Thirdly, LTCI financing criteria in Jiaxing align with Ningbo and Tonglu (90 Chinese Yuan (CNY)/person/year), reflecting the standards across five cities. Therefore, this study adopted Jiaxing’s LTCI criteria as the parameters of LTCI simulations in Zhejiang. The unit costs of HBDC, institutional care, HBDMC and nursing hospital care are set at 1200 CNY/month, 2100 CNY/month, 1680 CNY/month and 1680 CNY/month in 2024 according to LTCI maximum benefits in Jiaxing (see Additional Table 1, Additional file 1) [41]. The contributory group of LTCI is the group participating in social health insurance, whether retired or not. The LTCI financing parameter $premiu{m_{{t_0}}}$is based on a fixed amount of premiums in Jiaxing, of which the standard is 90 CNY/person/year [41].

Chinese government proposed a model of elderly care named “9073” model: 90% of older people receive home-based care, 7% receive community care and 3% receive institutional care [67]. “9073” model represents the prospects of China’s elderly care and is therefore suitable for the long-term simulation in this study [29, 62]. Specifically, proportion of HBDC (${\alpha _{\text{1}}}$), institutional care (${\alpha _2}$), and combination of HBDMC and nursing hospital care (${\alpha _3}$+${\alpha _4}$) are set at 90%, 3% and 7%, respectively. Disabled older people can choose to receive HBDMC at home or receive nursing hospital care at medical institutions when facing medical care needs. It is free to choose the locations for these two LTC services, and it is quite similar to receiving community care in nature, as it also allows the option of receiving services at home or at community centers. Additionally, the LTCI benefits of these two LTC services in Jiaxing are equal. Therefore, we grouped them together when determining the beneficiaries’ choice of LTC services type (${\alpha _3}$+${\alpha _4}$). We set the interest rate of LTCI fund at 2.5% based on current interest rate of 5-year time deposit in China’s banks [68]. The sources of each parameter for simulation framework of the study are demonstrated in Additional Fig. 1 (see Additional File 1).

## Results

### The mortality pattern and life expectancy of Zhejiang

**Fig. 1:**
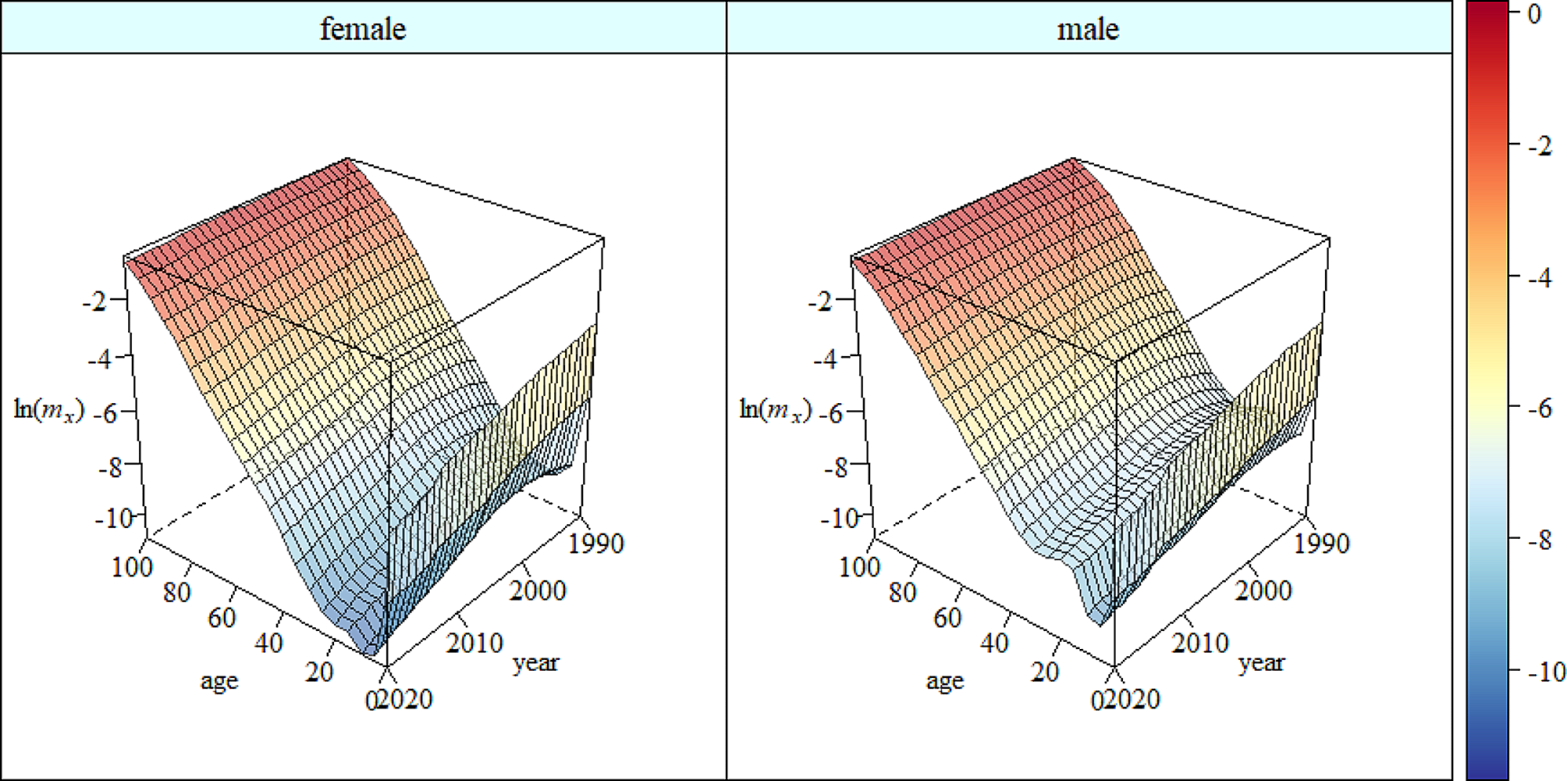
Mortality pattern of Zhejiang in 1990–2020 based on adjusted DCMD model

The estimated mortality of Zhejiang from 1990 to 2020 is demonstrated in Fig. 1 based on adjusted DCMD model. Overall, the mortality for male and female presents a declining trend. Specially, the child mortality had a continued decline during the estimation period, but the adult mortality and old-age mortality had a slight increase between 1990 and 2000, then with a sharp decline between 2000 and 2020 afterwards.

**Fig. 2:**
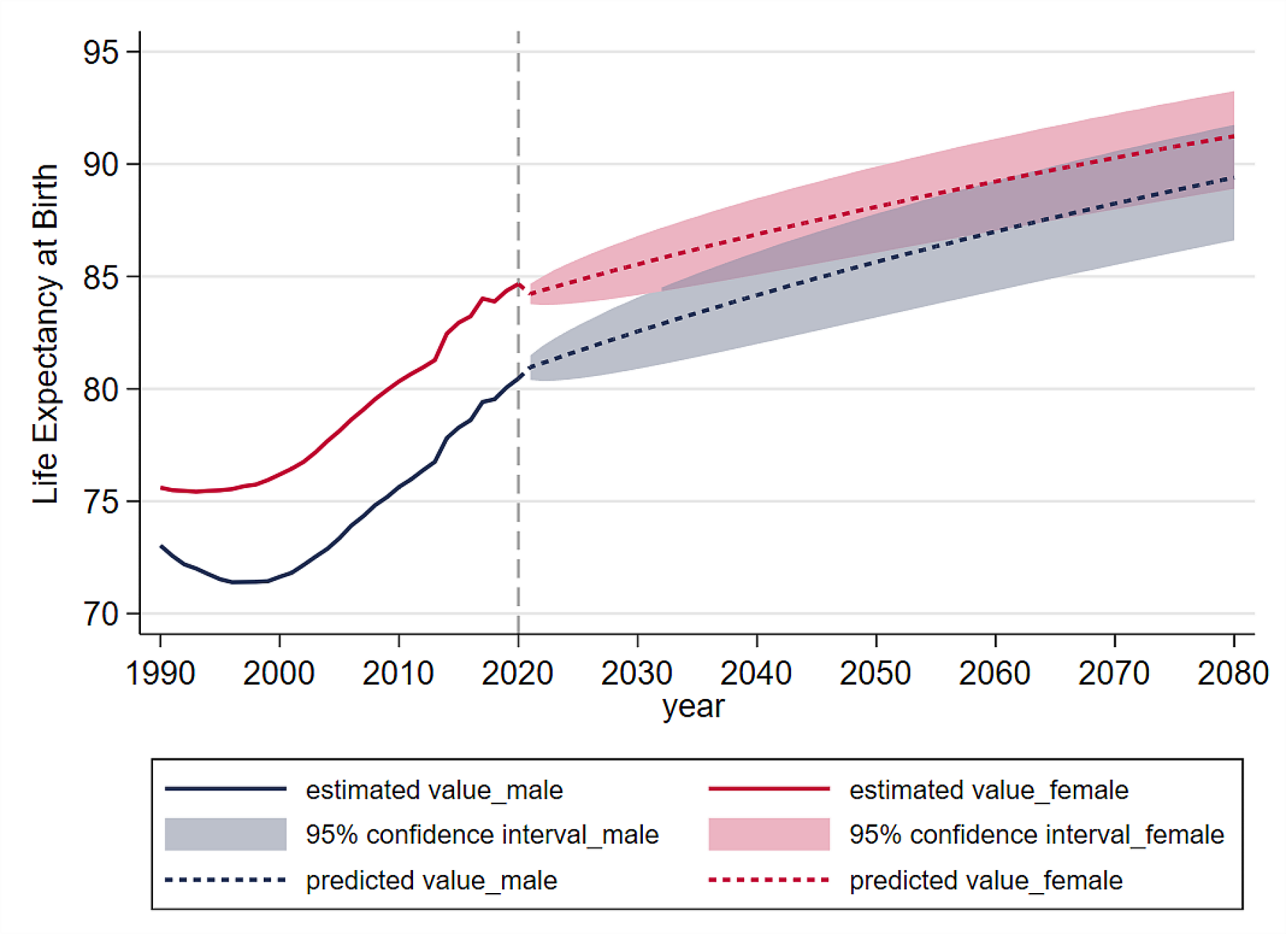
Estimated and predicted life expectancy of Zhejiang in 1990–2080

We further predict the life expectancy at birth with 95% confidential interval under the LC_ER model from 2020 to 2080. The estimated and predicted life expectancy is demonstrated in Fig. 2. Life expectancy of female had a stable increase from 1990 to 2020. While there was a slight decline of life expectancy of male from 1990 to 2000, then there was a rapid increase until 2020. The model results based on historical information show that life expectancy of both female and male will have an upward trend from 2020 to 2080. Besides, the gender difference in life expectancy will remain relatively stable in the future. In 2020, life expectancy was 80.46 years for male, 84.66 years for female. In 2080, the life expectancy will reach 89.39 [86.61, 91.74] years for male, 91.24 [88.90, 93.25] years for female. Besides, the age-specific rates of mortality decline of Zhejiang from 2021 to 2080 estimated by LC_ER are illustrated in Additional Fig. 2 (see Additional File 1).

### The simulation of long-term care demand and expenditures in Zhejiang

Based on CCP method, the study has projected the number of older people and the number of severely disabled older people with different scenarios of disability in Zhejiang from 2020 to 2080 (shown in Table 1). It is illustrated that the population aged 60 and above in Zhejiang will firstly expand to around 2060 and then shrink until 2080. The number of older people with disabilities, especially those with severe disability, reflects the long-term care demand from a demographic perspective. We found that the number of older people with severe disability will continue to increase to 2080 under both medium and high disability scenarios. However, the number of older people with different degrees of disability will increase before 2060, and then decline slightly in the following 20 years under the low disability scenario. We also found that the number of severely and moderately disabled older people will be of little difference before 2050, which means that severe and moderate LTC demand is roughly equal.

**Table 1:** Projection of the total older population with different degree of disability in Zhejiang under medium life expectancy scenario (in thousand people)

Year	Older population	With severe disability			With moderate disability			With mild disability		
		Medium disability scenario	High disability scenario	Low disability scenario	Medium disability scenario	High disability scenario	Low disability scenario	Medium disability scenario	High disability scenario	Low disability scenario
2023	14,625	285	295	276	288	297	279	1393	1438	1349
2030	19,266	386	418	356	383	414	353	1845	1998	1703
2040	24,738	592	694	504	571	669	486	2618	3071	2230
2050	29,089	810	1029	637	753	956	592	3229	4101	2538
2060	30,728	941	1295	683	848	1166	615	3544	4874	2570
2070	29,300	1000	1489	669	869	1294	582	3503	5218	2344
2080	27,417	1027	1657	634	858	1384	530	3389	5466	2093

*Notes* The older population is based on the predicted mean of life expectancy. High/Low disability scenarios represent that the growth/descent rate of age-specific prevalence rate of disability will be 0.8% per year. Medium disability scenario means the age-specific prevalence rate of disability will be fixed

Besides, the results of LTC demand under the high and low life expectancy scenarios are illustrated in Additional Table 2 and Additional Table 3 (see Additional file 1). It can be seen that Zhejiang Province will have a higher LTC demand under the scenario of higher life expectancy. The number of older people with severe disability under 95% upper interval of life expectancy in 2080 is 154 thousands higher than that under the predicted mean of life expectancy. And the number of older people with severe disability under 95% lower interval of life expectancy in 2080 is 169 thousands lower than that under the predicted mean of life expectancy. This result demonstrates the importance of mortality level prediction for assessing LTC demand.

**Fig. 3:**
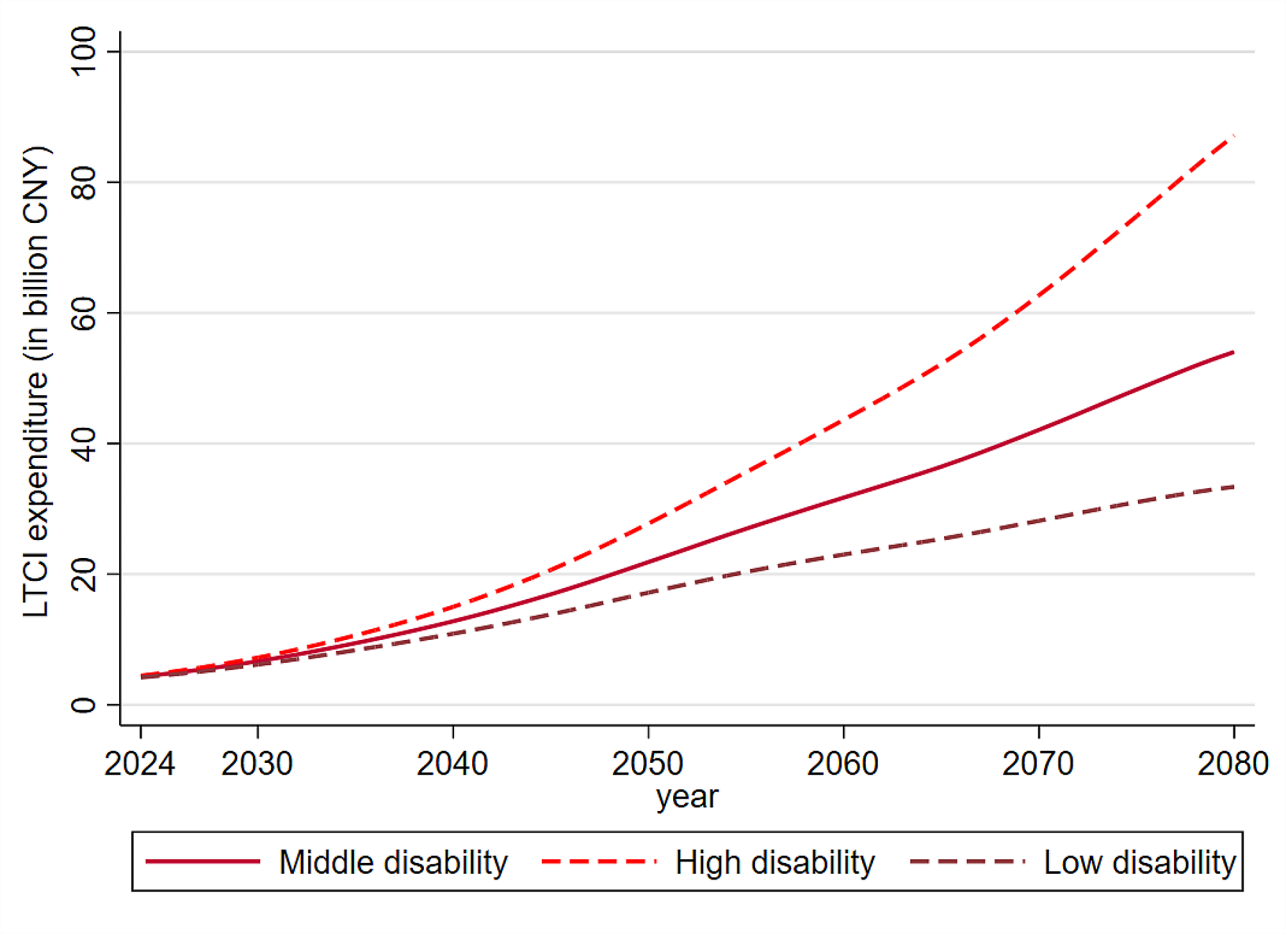
Projection of Long-term care insurance expenditure in Zhejiang, 2024–2080. *Notes* Results are based on the predicted mean of life expectancy

Our study further calculated the LTCI expenditure paid by insurance fund every year from 2020 to 2080 to analyze the future long-term care demand in our case area from a financial perspective. The expenditure from LTCI illustrates an upward trend from 2023 to 2080 (see Fig. 3), with the higher price of long-term care services and increasing number of severe disabled older people. The LTCI expenditure is still increasing although there will be a slight decline in severe disabled older people under low disability scenario.

### The simulation of LTCI fund under diverse disability and financing scenarios

**Fig. 4:**
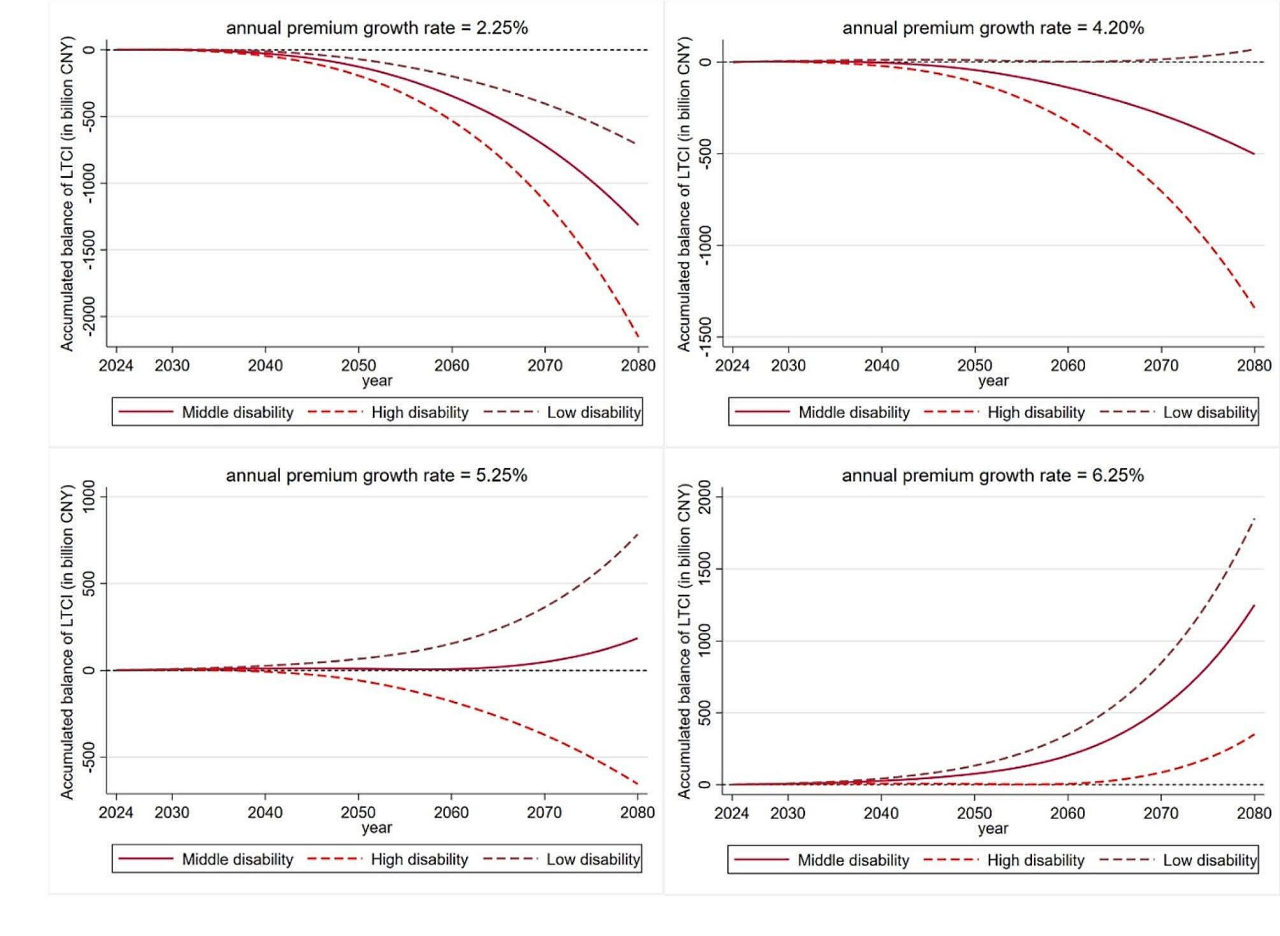
Accumulated balance of LTCI fund under different financing and disability scenarios. *Notes* Results are based on the predicted mean of life expectancy

**Fig. 5:**
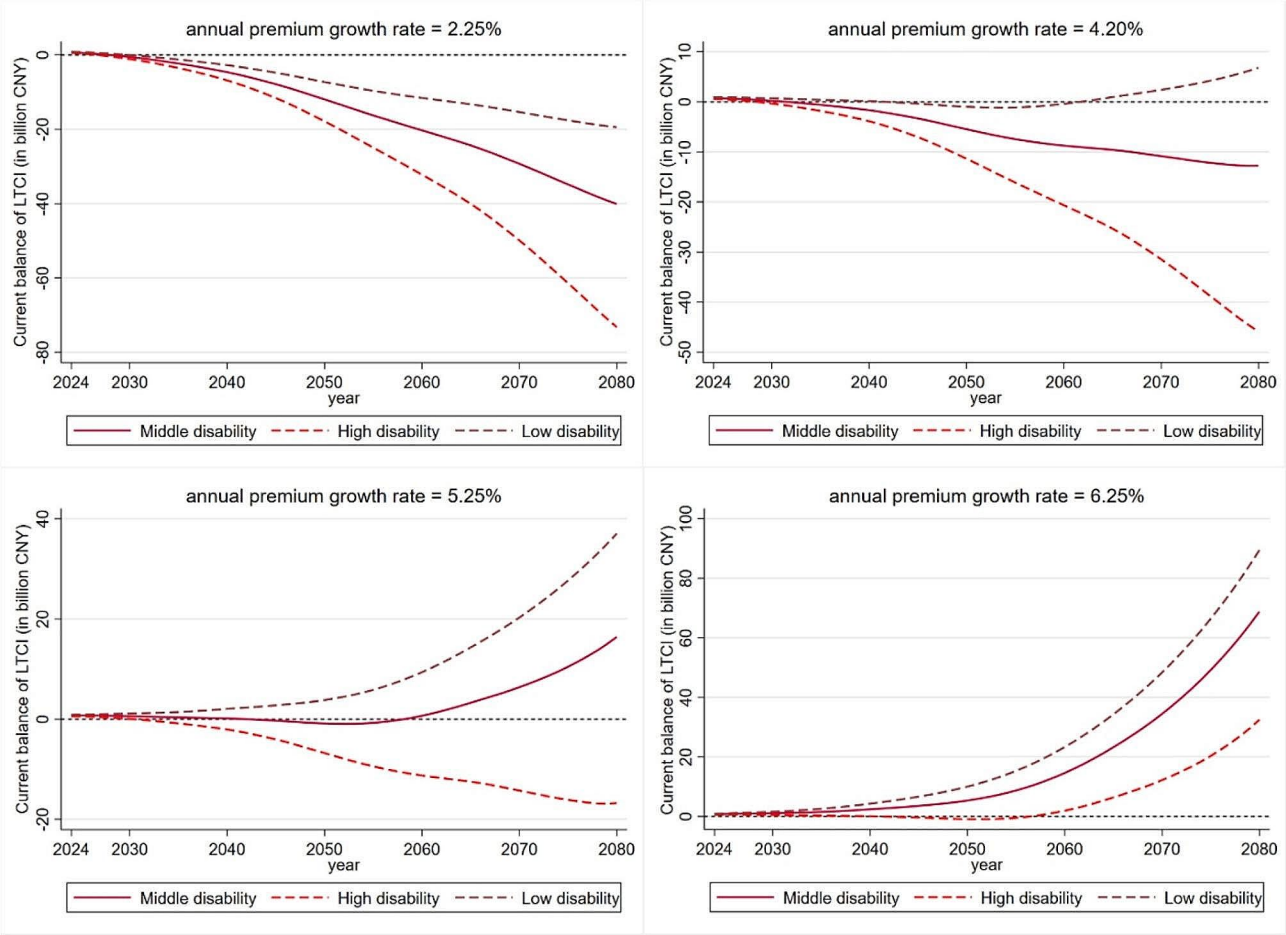
Current balance of LTCI fund under different financing and disability scenarios. *Notes* Results are based on the predicted mean of life expectancy

The accumulated balance of LTCI fund from 2022 to 2080 is simulated on different dynamic financing growth scenarios in order to test how to make LTCI achieve actuarial balance in the long run. The accumulated balance and current balance of LTCI fund in Zhejiang are shown in Figs. 4 and 5. When we set the annual premium growth rate at 2.25% which is equal to the average increase of healthcare CPI, there will be a deficit of current balance before 2028. As a result, the accumulated balance will become negative in 2032 under medium disability scenario, under high disability scenario in 2030 and under low disability scenario in 2036. This result shows that LTCI fund can only be sustainable within 12 years if the financing level grows at a low pace from 2024.

The minimum annual premium growth is further tested to achieve the positive accumulated balance of LTCI fund under various scenarios from 2022 to 2080. We found that when the annual premium growth rate equals to 4.20%, LTCI fund will realize the long-term sustainable under low disability scenario, which means that the 4.20% financing growth standard is effective to make LTCI sustainable at a relatively low premium level under low disability scenario; however, it will still face the risk of the shortage of financing with 4.20% annual premium growth under the medium and high disability scenarios after 2039 and 2033.

Furthermore, the accumulate balance of LTCI fund remains at a moderate surplus and will not face a shortage until 2080 under the medium disability scenario when the annual premium growth rate equals 5.25%. Although the current balance of LTC fund will be negative in 2043 to 2058 under 5.25% annual premium growth (see Fig. 5), the accumulated surplus before 2042 and continuous interest will still realize the accumulated surplus of LTCI fund (5.83 billion CNY) in 2058. Overall, the annual premium growth rate at 5.25% is the best parameter choice if the age-specific prevalence rate of disability in Zhejiang Province is projected to remain stable. Finally, LTCI will be sustainable under all disability scenarios when the premium increases by 6.25% per year. However, this level will put a heavy payment burden on the residents, and there will be a large amount of fund redundancy if the disability does not continue to increase.

### The simulation of LTCI fund under diverse life expectancy and financing scenarios

**Fig. 6:**
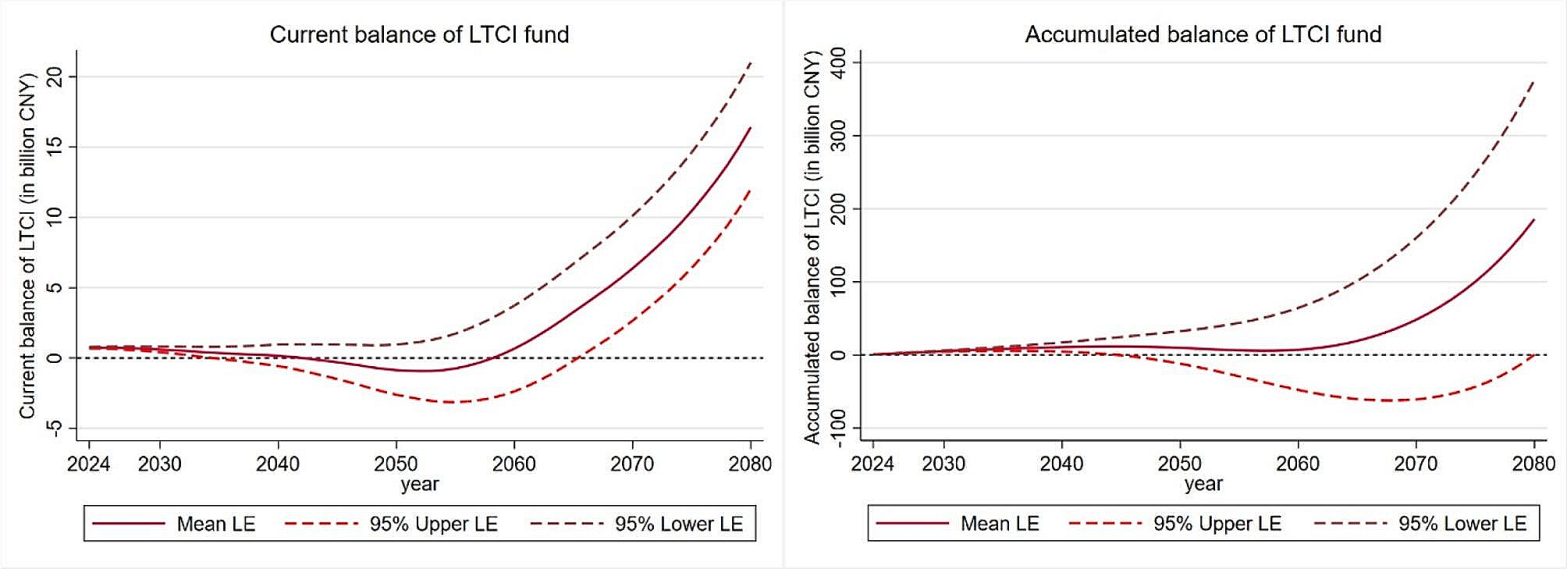
Current and accumulated balance of LTCI under different life expectancy scenarios. *Notes* Results are based on the 5.25% annual premium growth rate scenario and medium disability scenario

The impact of different life expectancy trend on the sustainability of LTCI schemes is further discussed. The simulation results of accumulated balance of LTCI fund under predicted mean, 95% upper confidential interval and lower confidential interval of life expectancy scenarios are demonstrated in Fig. 6. It is learned that the sustainability of the LTCI fund will face a completely different situation in the long-term because of the difference trends in life expectancy even under the same disability level and financing level. Under the 5.25% annual premium growth rate and medium disability scenario, LTCI fund will become accumulated deficit under 95% upper interval of life expectancy after 2045. However, the LTCI fund will always remain in surplus before 2080 with the predicted mean or lower 95% interval of life expectancy. Therefore, the balance of LTCI fund is sensitive to life expectancy. In addition to affecting LTC expenditures when other conditions are the same, life expectancy is also related the total amount of financing by the number of contributors, thereby influencing the sustainability of LTCI fund.

## Discussion

This study shows two novel contributions to the existing literature. The first contribution is that we have found an important but often overlooked point that LTCI financing is sensitive to the variability of life expectancy in the long-term. In 2080, the 95% upper interval of the life expectancy in Zhejiang Province will be 2.01 years for female (2.35 years for male) higher than the predicted mean, and its cumulative impact will make LTCI unsustainable 35 years in advance. This finding shows that the accurate estimation of life expectancy is critical for assessing the sustainability of social insurance schemes like LTCI [69, 70], and also reveals the significance of life expectancy analysis in this study, because health factors can be dynamically monitored through the evaluation and reimbursement records within the LTCI system [34, 71], but life expectancy estimation will become difficult due to the lack of timely statistical data. Besides, the study also finds that LTCI financing is also sensitive to the variability of prevalence rate of disability in the long-term. Only 4.20% annual growth of premium can make Zhejiang’s LTCI sustainable under a disability compression assumption. However, the 6.25% annual growth of premium is necessary for Zhejiang’s LTCI sustainability under disability expansion assumption. The results are consistent with some existing research with various disability scenarios [28, 72]. The overall incidence of disability will face a growing trend with population aging [17]. Therefore, proposing health promotion and postponing disability actions to reduce the incidence and duration of severe disability among older people will mitigate the pressure of LTCI funding [73].

The second contribution is that Zhejiang’s LTCI financing needs to grow at a relative high speed annually (5.25% under the medium scenario) to achieve sustainability in the long-term. It should be noticed that the LTCI financing parameters to achieve short-term and long-term fund equilibrium are different, and it is clear that long-term fund balance is a necessary condition to ensure the sustainability of the system [22, 29]. If the accumulated surplus of the LTCI fund in Zhejiang Province before 2050 is used as a criterion for determining sustainability, as many studies have done [19, 74], our results indicate that Zhejiang LTCI fund is projected to experience an accumulating deficit for over 20 years after 2050. Like Zhejiang, there are also several pilot cities in China that have adopted the fixed amount of premium model without premium adjustment [32]. LTCI funds in these regions will run the risk of accumulating deficits in the short term [43]. China and other countries adopting social LTCI need to adjust the scale of premium in a timely and dynamic manner to cope with the long-term LTCI financing pressure since China’s aging plateau will continue after 2060 [47].

Our simulation results can also be used as a reference for countries and regions that adopt a fixed percentage of income model of LTCI financing although we focus on the fixed amount model of LTCI financing. The study finds that LTCI premium in Zhejiang needs to increase by 5.25% per year to ensure sustainability to 2080 under the assumption of disability with dynamic equilibrium. However, the growth rate may exceed the income growth rate of some countries in the context of declining global economic growth [75]. Therefore, even those countries based on a fixed percentage of income model need adjust financing parameters dynamically [1]. In LTCI fund management, China and other countries can learn from Germany’s experience to deal with the long-term impact of population aging, which has established a demographic reserve fund which saves 0.1% of premium every year for payment in the future [25].

Reasonable coverage and benefits are also important factors to achieve sustainable LTCI. Like developed countries, the LTCI pilot cities in Zhejiang Province cover all urban and rural residents. However, most of the LTCI pilot cities in China only cover urban employees [35]. Therefore, the analysis of LTCI in Zhejiang Province in this paper provides implications for other LTCI pilot cities in China to expand the coverage and promote the equity of receiving LTC. Besides, it should be noted that this study only considers the older adults with severe disabilities according to the rules when estimating LTC needs in Zhejiang Province [36]. Whereas, it is not only the families of severely disabled groups that face the burden of long-term care [17]. Moderately disabled people in some developed countries and pilot cities in China are also covered by LTCI [76, 77]. Even considering only severe disability, our simulation results show that only a high premium growth rate can make the system sustainable in the long run. Therefore, LTCI policymakers need to comprehensively weigh residents’ payment pressure and long-term care benefits, and make a balance between expanding coverage and increasing financing with the aim of protecting the most vulnerable groups.

This study has explored and built a long-term care insurance system that can be a reference for China and other developing countries to provide LTC services for the disabled older adults in the future. The strength of this study is that a more accurate life expectancy estimation based on the DCMD model is adopted when estimating dynamic financing of LTCI. However, this paper still has some limitations. Firstly, the paper only considers the activities of daily living when estimating the prevalence rate of disability of older people in Zhejiang Province, but does not consider cognitive function, perception and communication function due to the lack of data. Secondly, this study only considers the expenditure cost of LTC in the simulation analysis, but does not consider the operating cost of the LTCI system. Thirdly, this study only considers the total amount of financing for LTCI, but does not discuss the financing structure including individual contributions, government subsidies, and pooling funds. Finally, this study focuses only on the case in Zhejiang, but does not simulate the LTCI financing standard for actuarial equilibrium in other LTCI pilot areas in China.

## Conclusion

In summary, this study estimates and predicts the mortality rate in Zhejiang Province from 1990 to 2080 through the DCMD model and LC model, and further evaluates the increasing LTC need in Zhejiang Province in the future. The LTCI dynamic financing in Zhejiang Province under different disability scenarios and life expectancy scenarios is simulated on the LTCI expenditure forecast results, and it is found that only by maintaining a relatively high level (5.25% under medium scenario) of premium growth can Zhejiang’s LTCI be sustainable in the long run. Our empirical case in Zhejiang offers implications for developing countries and LTCI pilot areas that lack high-quality mortality information to establish and dynamically optimize LTCI financing. Therefore, policy makers are called upon to assess the sustainability of LTCI from a long-term perspective, and regularly monitor changes in residents’ health and life expectancy to ensure that LTCI fund can meet LTCI expenditure and control the financing burden.

### Electronic supplementary material

Below is the link to the electronic supplementary material.

Supplementary Material 1

## Data Availability

In this study, all the data sources are publicly available. The data calculated in this study is available upon request to the corresponding author.

## Abbreviations

LTC: Long-term care
LTCI: Long-term care insurance
CHARLS: China Health and Retirement Longitudinal Study
CDC: Center for Disease Control and Prevention
CPI: Consumer Price Index
DCMD: Developing Country Mortality Database
LC_ER: Lee-Carter method extended with rotation
CCP: Cohort-component projection
CNY: Chinese Yuan
HBDC: Home-based daily living care
HBDMC: Home-based daily living & medical care
