# Supplementary material for: Life expectancy, long-term care demand and dynamic financing mechanism simulation: an empirical study of Zhejiang Pilot, China
Source: BMC Health Serv Res. 2024 Apr 15;24:469. doi: 10.1186/s12913-024-10875-7 (PMC11017606; doi:10.1186/s12913-024-10875-7)
Supplement: Supplementary file 1 — Supplementary Material 1 [file 12913_2024_10875_MOESM1_ESM.docx]

# Additional file 1


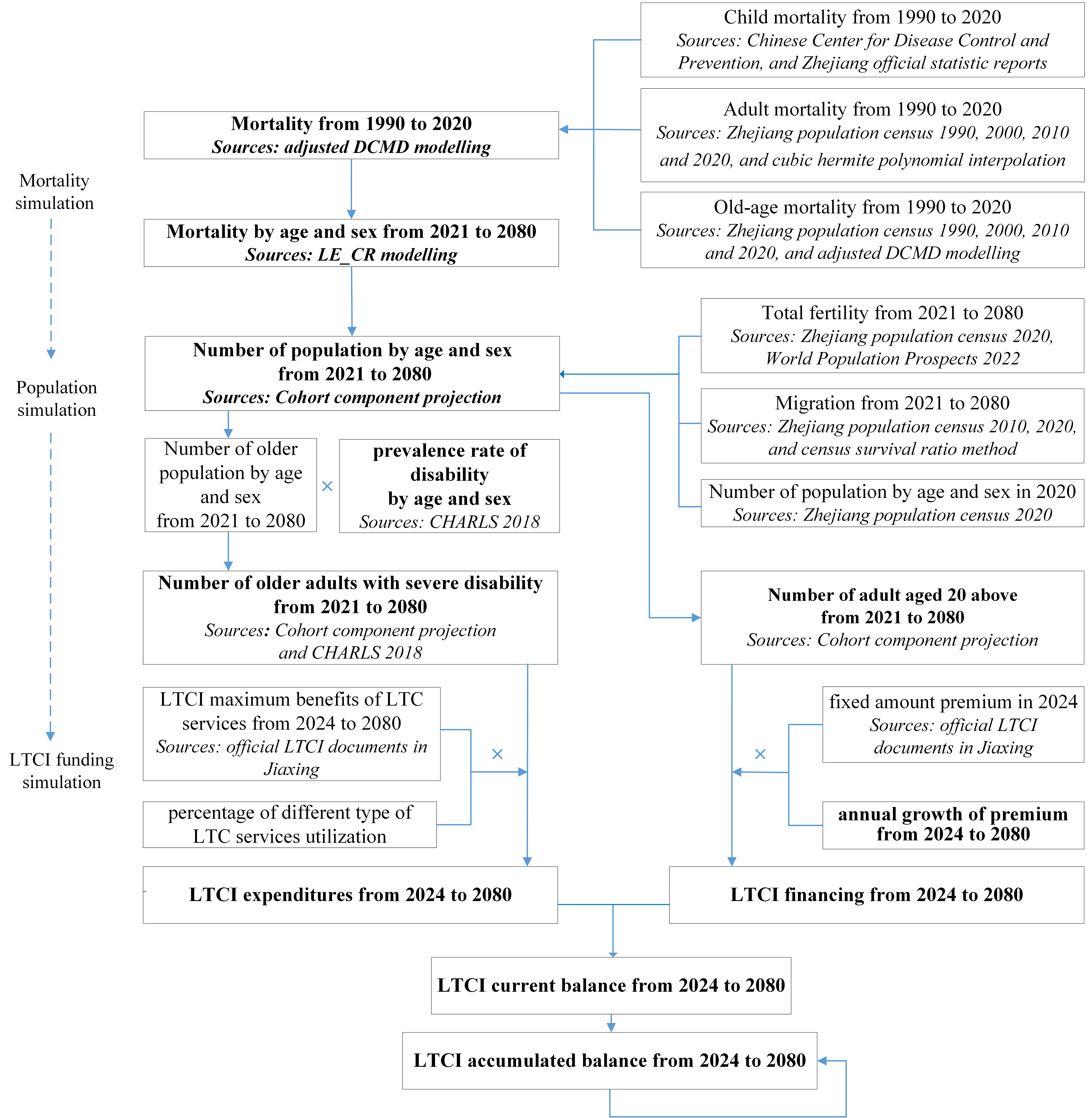


Additional Figure 1 Simulation framework of Zhejiang LTCI dynamic financing model


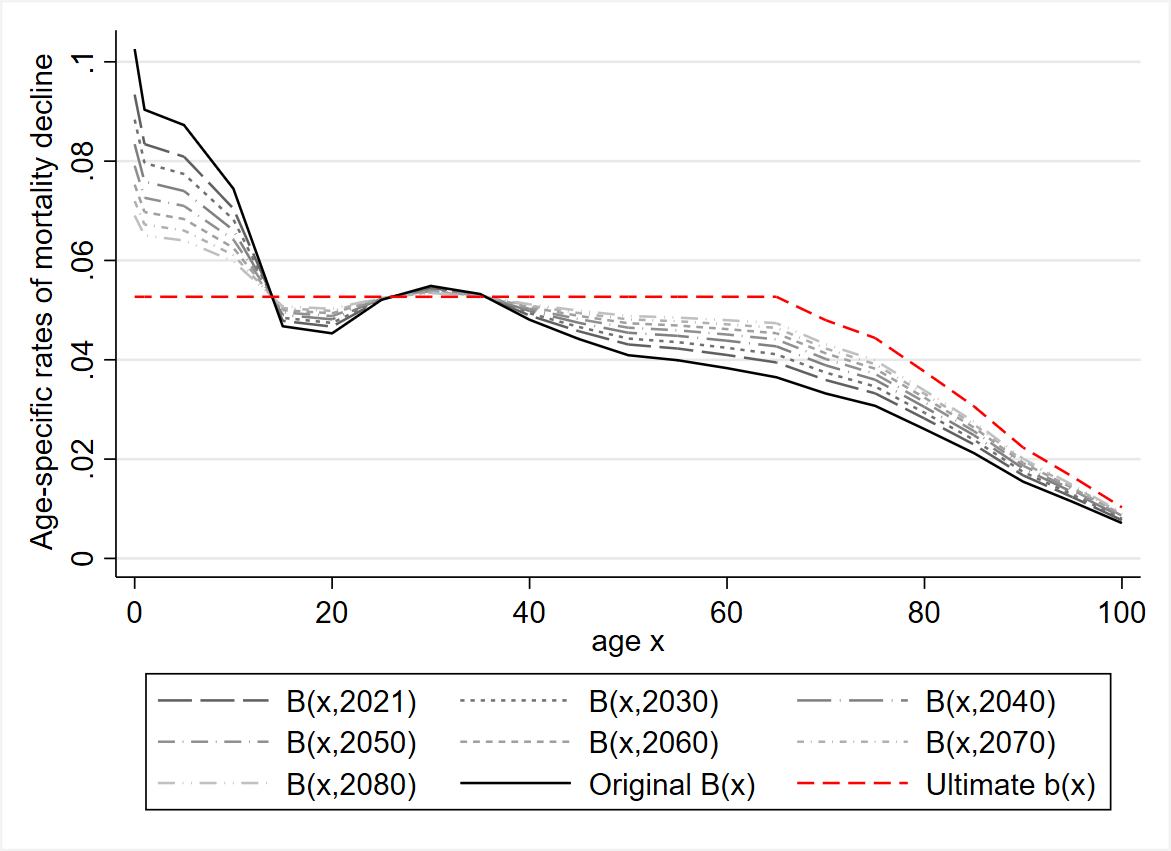


Additional Figure 2 Time-varying of age pattern of mortality decline from 2021 to 2080 based on LC_ER model

Notes: B(x,t) means the age pattern of mortality decline with x ages in t years. Original B(x) means the original values of age pattern of mortality decline by Lee-Carter coherent model. Ultimate b(x) means the ultimate values of the rotation of age pattern of mortality decline defined by Li et al. (2013).

Additional Table 1 Financing and benefit criteria of LTCI between 5 piloted cities in Zhejiang

| city | financing criteria | payment limit (LTCI payment percentage) | maximum benefit of LTCI (CNY/month)^**^ | When implementing LTCI | When covered residents |
| --- | --- | --- | --- | --- | --- |
| Jiaxing | 90 CNY/person/year | home-based daily living care: 1500 CNY/month (80%);  home-based daily living & medical care: 2100 CNY/month (80%);  nursing hospital care: 2400 CNY/month (70%);  institutional care: 3000 CNY/month (70%) | home-based daily living care: 1200;  home-based daily living & medical care: 1680;  nursing hospital care: 1680;  institutional care: 2100 | 2017 | 2017 |
| Yiwu | employees: 2.5‰ of personal wages;  residents: 2‰ of annual disposable income per capita | home-based care: 90 CNY/day (80%);  nursing hospital care: 150 CNY/day (70%);  institutional care: 100 CNY/day (70%) | home-based care: 2160;  nursing hospital care: 3150;  institutional care: 2100 | 2018 | 2018 |
| Wenzhou | employees: 100 CNY/person/year  residents: 90 CNY/person/year | home-based care: 1788-2980 CNY/month (80%)^*^;  institutional care: 1788-2980 CNY/month (80%)^*^;  informal care subsidy: 894-1490 CNY/month^*^ | home-based care: 1430.4-2384;  institutional care: 1430.4-2384;  informal care subsidy: 894-1490 | 2019 | 2023 |
| Ningbo | 90 CNY/person/year | home-based care: 1300-1950 CNY/month (80%)^*^  fixed payment of institutional care: 40-60 CNY/day^*^ | home-based care: 1040-1560;  institutional care: 1200-1800 | 2017 | 2022 |
| Tonglu | 90 CNY/person/year | fixed payment of nursing-home care: 40 CNY/day  fixed payment of institutional care: 30 CNY/day  home-based care: 1200-1500 CNY/month (80%)^*^ | nursing-home care: 1200;  institutional care: 900;  home-based care: 1200-1500 | 2017 | 2022 |

Notes: Information is compiled as of Jan 2024. ^*^ The interval of LTCI benefit was for beneficiaries with different group of severe disability. Severe disability is further defined as severe Ⅰ level, severe Ⅱ level and severe Ⅲ level according to standards set by each pilot city, to differentiate benefit levels for beneficiaries. ^**^ We convert daily benefits to monthly benefits based on 30 days in a month.

Additional Table 2 Projection of the number of total older population with different degree of disability in Zhejiang under high life expectancy scenario (in thousand people)

| year | Older  population | with severe disability | | | with moderate disability | | | with mild disability | | |
| --- | --- | --- | --- | --- | --- | --- | --- | --- | --- | --- |
|  |  | medium  disability  scenario | high  disability  scenario | low  disability  scenario | medium  disability  scenario | high  disability  scenario | low  disability  scenario | medium  disability  scenario | high  disability  scenario | low  disability  scenario |
| 2023 | 13990 | 275 | 282 | 268 | 277 | 284 | 271 | 1337 | 1370 | 1305 |
| 2030 | 19612 | 400 | 433 | 369 | 394 | 427 | 364 | 1890 | 2047 | 1744 |
| 2040 | 25674 | 636 | 746 | 541 | 606 | 711 | 516 | 2751 | 3227 | 2343 |
| 2050 | 30682 | 896 | 1138 | 704 | 820 | 1041 | 644 | 3460 | 4394 | 2719 |
| 2060 | 32826 | 1068 | 1470 | 775 | 939 | 1292 | 681 | 3846 | 5290 | 2789 |
| 2070 | 31537 | 1140 | 1699 | 763 | 967 | 1440 | 647 | 3805 | 5667 | 2546 |
| 2080 | 29683 | 1181 | 1905 | 729 | 958 | 1545 | 592 | 3702 | 5972 | 2287 |

Notes: The number of older population is based on the upper 95% predicted interval of life expectancy. High/Low disability scenarios represented the growth/descent rate of age-specific prevalence rate of disability will be 0.8% per year. Medium disability scenario means the age-specific prevalence rate of disability will be fixed.

Additional Table 3 Projection of the number of total older population with different degree of disability in Zhejiang under low life expectancy scenario (in thousand people)

| year | Older  population | with severe disability | | | with moderate disability | | | with mild disability | | |
| --- | --- | --- | --- | --- | --- | --- | --- | --- | --- | --- |
|  |  | medium  disability  scenario | high  disability  scenario | low  disability  scenario | medium  disability  scenario | high  disability  scenario | low  disability  scenario | medium  disability  scenario | high  disability  scenario | low  disability  scenario |
| 2023 | 13854 | 270 | 276 | 263 | 273 | 279 | 266 | 1320 | 1352 | 1288 |
| 2030 | 18879 | 371 | 402 | 342 | 370 | 401 | 342 | 1796 | 1945 | 1657 |
| 2040 | 23661 | 544 | 638 | 463 | 531 | 623 | 452 | 2467 | 2894 | 2101 |
| 2050 | 27277 | 718 | 912 | 564 | 680 | 863 | 534 | 2968 | 3769 | 2332 |
| 2060 | 28373 | 809 | 1112 | 586 | 748 | 1028 | 542 | 3200 | 4402 | 2321 |
| 2070 | 26755 | 851 | 1268 | 570 | 760 | 1133 | 509 | 3146 | 4686 | 2105 |
| 2080 | 24758 | 858 | 1383 | 530 | 741 | 1195 | 458 | 3002 | 4843 | 1854 |

Notes: The number of older population is based on the lower 95% predicted interval of life expectancy. High/Low disability scenarios represented the growth/descent rate of age-specific prevalence rate of disability will be 0.8% per year. Medium disability scenario means the age-specific prevalence rate of disability will be fixed.
